# Retraction: lncRNA HOTAIR promotes gastric cancer proliferation and metastasis via targeting miR‐126 to active CXCR4 and RhoA signaling pathway

**DOI:** 10.1002/cam4.7220

**Published:** 2024-05-02

## Abstract

Retraction: Jun Xiao, Hao Lai, Sheng‐Hong Wei, Zai‐Sheng Ye, Fu‐Sheng Gong, Lu‐Chuan Chen (2019), lncRNA HOTAIR promotes gastric cancer proliferation and metastasis via targeting miR‐126 to active CXCR4 and RhoA signaling pathway. Cancer Med, 8: 6768–6779. https://doi.org/10.1002/cam4.1302

The above article, published online on 13 September 2019, and a corresponding Corrigendum (https://doi.org/10.1002/cam4.4435), published online on 22 November 2021, in Wiley Online Library (wileyonlinelibrary.com) have been retracted by agreement between the journal's Editor‐in‐Chief, Stephen Tait, and John Wiley & Sons Ltd. Following publication, concerns were raised by a third party regarding duplication of images included within Figures 3C and 5E. Furthermore, these figures have previously been published elsewhere, identifying the cells as originating from different cell lines and subjected to different treatment. The authors did not respond to the concerns raised and did not provide the original data. The editors consider the results and conclusion reported in this article unreliable.

Retraction: Jun Xiao, Hao Lai, Sheng‐Hong Wei, Zai‐Sheng Ye, Fu‐Sheng Gong, Lu‐Chuan Chen (2019), lncRNA HOTAIR promotes gastric cancer proliferation and metastasis via targeting miR‐126 to active CXCR4 and RhoA signaling pathway. Cancer Med, 8: 6768–6779. https://doi.org/10.1002/cam4.1302

The above article, published online on 13 September 2019, and a corresponding Corrigendum (https://doi.org/10.1002/cam4.4435), published online on 22 November 2021, in Wiley Online Library (wileyonlinelibrary.com) have been retracted by agreement between the journal's Editor‐in‐Chief, Stephen Tait, and John Wiley & Sons Ltd. Following publication, concerns were raised by a third party regarding duplication of images included within Figures 3C and 5E. Furthermore, these figures have previously been published elsewhere, identifying the cells as originating from different cell lines and subjected to different treatment. The authors did not respond to the concerns raised and did not provide the original data. The editors consider the results and conclusion reported in this article unreliable.

